# Palliative care in the neonatal unit: neonatal nursing staff perceptions of facilitators and barriers in a regional tertiary nursery

**DOI:** 10.1186/s12904-017-0202-3

**Published:** 2017-05-11

**Authors:** Meegan Kilcullen, Susan Ireland

**Affiliations:** 1 College of Healthcare Sciences, James Cook University, Townsville, QLD 4811 Australia; 2 Department of Neonatology, The Townsville Hospital, Townsville, QLD 4811 Australia; 3 College of Medicine and Dentistry, James Cook University, Townsville, QLD 4811 Australia

**Keywords:** Palliative care, Neonatal, Facilitators, Barriers, Regional location, Qualitative

## Abstract

**Background:**

Neonatology has made significant advances in the last 30 years. Despite the advances in treatments, not all neonates survive and a palliative care model is required within the neonatal context. Previous research has focused on the barriers of palliative care provision. A holistic approach to enhancing palliative care provision should include identifying both facilitators and barriers. A strengths-based approach would allow barriers to be addressed while also enhancing facilitators. The current study qualitatively explored perceptions of neonatal nurses about facilitators and barriers to delivery of palliative care and also the impact of the regional location of the unit.

**Methods:**

The study was conducted at the Townsville Hospital, which is the only regional tertiary neonatal unit in Australia. Semi-structured interviews were conducted with a purposive sample of eight neonatal nurses. Thematic analysis of the data was conducted within a phenomenological framework.

**Results:**

Six themes emerged regarding family support and staff factors that were perceived to support the provision of palliative care of a high quality. Staff factors included *leadership, clinical knowledge, and morals, values, and beliefs*. Family support factors included *emotional support, communication,* and *practices* within the unit. Five themes emerged from the data that were perceived to be barriers to providing quality palliative care. Staff perceived *education, lack of privacy, isolation, staff characteristics* and *systemic (policy, and procedure)* factors to impact upon palliative care provision. The regional location of the unit also presented unique facilitators and barriers to care.

**Conclusions:**

This study identified and explored facilitators and barriers in the delivery of quality palliative care for neonates in a regional tertiary setting. Themes identified suggested that a strengths-approach, which engages and amplifies facilitating factors while identified barriers are addressed or minimized, would be successful in supporting quality palliative care provision in the neonatal care setting. Study findings will be used to inform clinical education and practice.

## Background

Neonatology has made significant advances in the last 30 years. Surfactant therapy, improved ventilators and ventilation strategies, improved surgical techniques and parenteral feeding have enabled the survival of vulnerable babies [1, 2]. Despite the advances in treatments, not all neonates survive and a palliative care model is required within the neonatal context. Death on the neonatal unit may occur when intensive care support is withdrawn, there is a conscious limitation to the escalation of intensive care, or the baby cannot be kept alive despite all attempts to continue care [3, 4]. Australian data suggests that three quarters of deaths in the neonatal context occur after intensive care is withdrawn [5]. US data shows similarly high levels of withdrawal as a mode of death, particularly in babies with congenital anomalies, whilst withholding care is more common in extremely preterm babies.

The aims of palliative care in the neonatal context are to prevent and relieve pain and suffering of neonates and provide support for families. Such care includes planning with the family about the practicalities of the death and continuing family support after the baby dies [6, 7]. The timing of withdrawal must allow time for parents to prepare for the death of the baby but be balanced against the suffering of the baby [8]. The obligations for nurses and doctors is to provide options for parents, preparing them for the death, providing physical support to the family while providing comfort to the baby, advocating for the family and providing emotional support [8]. The basic elements for palliative care include the need for warmth, dignity, human contact and pain relief for the neonate and neonatal nurses are at the forefront of such care in the neonatal unit.

Limited research has been conducted that explores neonatal nurses’ perspectives of providing palliative care [9–13]. A systematic review identified “attitudinal, clinical, educational, institutional, regulatory, and financial” barriers to providing palliative care [10]. Specifically, barriers included nurses' values and moral dilemmas, beneficence and nonmaleficence, nurses' exposure to death, emotional control and protection, stress, grief, lack of optimal environment, and lack of education in palliative care principles. A subsequent Australian study identified facilitators of quality palliative care that included a health care team which is supportive of each member’s opinions and beliefs, availability of counseling for caregivers, the use of clinical guidelines and the provision of adequate support for parents [12]. Barriers were found to be a poor physical environment, technological imperatives and parental demands to continue treatment and concerns about harming the infant or contributing to suffering [10]. Similarly, barriers included the negative impact of lack of education including ineffective communication, and the assessment of needs and implementation of palliative care including a lack of clinical guidelines for providing palliative care [13].

Other research in the Australian context identified barriers to palliative care in neonatal nursing related to staffing, the environment and the technological imperatives [11]. Inadequate staffing was identified where the labour intensive nature of palliative care was not acknowledged by the organizational structures and insufficient staff was available to help nurses providing the care. The environment of the unit negatively impacted care when the physical structure was inadequate and privacy and comfort lacking for families. Additionally, moral distress was reported by nurses when they perceived an escalation of treatment via the use of technology in a futile situation. Moral distress was a result of treating a neonate with no hope of survival and contributing to false expectations of the parents. Moral distress has also been identified when nurses perceived continued intensive care was being provided which was not in the best interests of the neonate [13].

Chen and colleagues [9] in Taiwan, used a questionnaire approach to explore the attitudes and beliefs of neonatal nurses towards the dying neonate and to determine the influence of these on nurses’ attitudes towards palliative care. Similar to other studies [10, 11], barriers to quality palliative care included the lack of information to the parents about their options for palliative care, and nurses’ perception that they were not permitted to voice opinions about palliation. Nurses perceived a lack of resources and also having little palliative care education or guidelines for providing care. A lack of education for nurses has also been noted in the Australian [11, 12] and United States contexts [13]. The nurses perceived an overuse of technology to keep babies alive and parental opposition to palliation [12]. Cultural influences were noted in this study including a majority of participants who believed in transmigration of the soul, and a third who believed that palliative care was inappropriate as neonates are at the beginning of life [9].

Cultural implications of palliative care have been identified within New Zealand Maori and Australian Indigenous communities [14, 15]. While these studies were not in the neonatal context, important considerations for delivering culturally responsive palliative care are highlighted. For example, there are cultural considerations regarding the level of inclusion of family members in planning palliative care, which family members are appropriate to consult [15], and the impact of perceptions of death and dying and intervening in these processes [9, 14]. As others have identified [9], it is important to consider the influence of cultural influences upon perceptions of providing palliative care particularly at the beginning of life.

A holistic approach to enhancing palliative care provision should include identifying both facilitators and barriers. Previous research has focused on the barriers of palliative care provision [9–13]. A strengths-based approach would allow barriers to be addressed while also enhancing facilitators of palliative care. It is also important to note that previous studies have also relied on focus groups [12], secondary analysis [10, 11, 13] or questionnaire data [9] with little individual qualitative interview-based studies conducted. The current study qualitatively explored perceptions of neonatal nurses about facilitators and barriers that impact upon the delivery of palliative care. Such information is key to planning, implementing and evaluation strategies to harness facilitators and reduce effect of barriers in delivery of quality care. Further, the study explored the impact of regional location of the unit upon delivery of quality palliative care.

## Method

The study was conducted at The Townsville Hospital (TTH), Australia. TTH Neonatal Unit is the only regional tertiary unit in Northern Australia and has an extensive rural, remote and extremely remote catchment area. It serves an area of approximately 500 000 sq. km. Babies are also referred for surgery, excluding cardiac surgery. In 2015, 255 patients were admitted for intensive care, with 7 deaths from complications of prematurity, congenital anomalies, infections or hypoxic ischaemic brain injury. Approximately 75% of neonates are inborn and 25% are retrieved from areas across North Queensland. The study was conducted within a phenomenological framework that seeks to understand individuals ‘lived experience’ of providing palliative care in a neonatal unit.

### Participants

A purposive sample of eight neonatal nurses with experience in providing palliative care participated in the study. Eligibility criteria included part-time and full-time neonatal nurses who had experience providing palliative care in the neonatal context. The participating nurses had more than 5 years of neonatal nursing experience, and were registered nurses. They represent a varied skill mix. No further demographic information was collected.

### Materials

Interviews were guided by open-ended questions regarding the delivery of palliative care in a neonatal and regional context. Nurses were asked about their perceptions of barriers and facilitators of palliative care in the unit, and whether the unit’s regional location impacted upon the delivery of palliative care. Questions included “What is ‘end-of-life- care?”; “What is good ‘end-of-life’ care?”; “What promotes good ‘end-of-life’ care?”; “What do you think we do well on this Unit?”; and “Do you think our Unit being in a regional area affects our palliative care?”. After completion of the each interview, participants were offered an opportunity to add any further information about their experiences of neonatal palliative care in order to capture further relevant information.

### Procedure

The study was promoted via an email through the Nurse Unit Manager to all nursing staff and snowball recruiting was used to encourage participation. Semi-structured interviews were conducted with nurses at a location of their choice. Verbal and written consent was also obtained to conduct and digitally audio record the interview. Data was analysed within an Interpretative Phenomenological Analysis (IPA) framework [16]. IPA is a qualitative research methodology which describes the ‘lived experience’ in order to understand people’s perceptions of the study subject. Within the IPA framework, a 6 stage exploratory thematic analysis process was conducted as described by Braun and Clarke [17]. The thematic analysis was conducted using an iterative process in order to develop codes, categories, subcategories and themes. Results of this study meet Yardley’s qualitative research validity criteria of 1) sensitivity to context; 2) commitment and rigour; 3) transparency and coherence; and 4) impact and importance (see [16] for review of IPA and validity criteria). Themes were Ethics approval for the study was obtained from the local human research ethics committee (13QTHS84).

## Results

Results are presented within a framework of the facilitators and barriers of quality palliative care, followed by the effects of regional location and culture on palliative care.

### Facilitators of good care

**Fig. 1:**
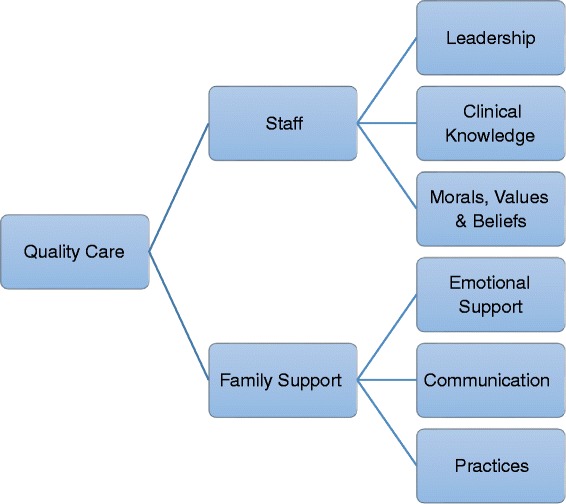
Facilitators of quality palliative care – staff and family support themes

Six themes were identified regarding family support and staff factors that were perceived to support the provision of quality palliative care. Staff themes included *leadership, clinical knowledge, and morals, values, and beliefs*. Family support themes included *emotional support, communication,* and *practices* within the unit (See Fig. 1). Each of these themes includes subthemes that impact upon the delivery of quality palliative care.

### Staff attributes

#### Leadership

Within the *leadership* theme, 6 subthemes were identified –*staff suitability and experience, mentorship, communication between staff members, skill mix, and supporting staff*. Quality palliative care was delivered when nursing leadership in the unit understood the interrelated nature of these factors when allocating and supporting staff during the palliative period for an infant and family. For example, leadership recognition of staff who were ***suitable and experienced*** in delivering palliative care was facilitated by those who self-identified as one nurse reported “I mean you have to be the type of person who will actually handle that and will be able to facilitate it and look after mentally [self and family]” (P 8). Further, staff were considered suitable and experienced when “people who sort of do read things and don’t shy away from the opportunities…and if they seem to be continue to be interested then you know that they’re not shy of it” (P2).

Leadership in the palliative care also reflected the positive influence of ***mentorship*** of staff during the palliative period. Mentorship was described by nurses as a collaborative way in which to engage with more experienced staff and to navigate their own emotional experiences during the palliative process.

My mentorship [as clinical lead nurse] with someone else if they’re doing end-of-life is just being able to provide that staff member with anything they need. You know, if they’re doing the end-of-life care and I’m just supporting them as a team lead, “what do you need?” (P2)

Effective leadership in the palliative period also recognised the ***skill mix*** of the staff. Nurses perceived that good leaders were able to identify the skill mix appropriate for both staff and family needs during this period. As one nurse reported, “I think sometimes we do really well like where we do think about the families and allocations” (P5).

Effective leadership facilitated clear ***communications*** between staff members during the palliative period to enhance care. Clear communication allowed staff members to provide integrated and collaborative care to the infant and families. As one nurse noted:

Communication, most definitely…between medical, whatever hospital staff are involved. Definitely between the family and Allied, and I’m talking Allied Health too, whatever hospital people are involved that needs… you and whoever, social worker or whatever. (P3)

#### Clinical knowledge

Within the theme of *clinical knowledge*, 3 subthemes emerged – *education, adapting and tailoring care, and medical support*. Respondents reported that palliative care was facilitated by the depth of clinical knowledge of nursing staff through ongoing ***education***. This educative process supported staff to ***adapt*** and ***tailor*** care to infants and families’ needs. As one nurse reported:

I think mostly we’ve got a unit where there’s a lot of knowledge around and so we can inform the parents, consultants down too, but mostly the senior nurses I’d say. We’ve got a lot of junior ones but we’ve got a lot of knowledge about what can happen and explain to parents what can happen. (P1)

Nurses also acknowledged the family-centred ***medical support*** by consultants during the palliative care period. For example, one nurse reported that consultants were good at “explaining things easy for them to understand” (P7), however, the hierarchy was also noted:

you know what they, they obviously dictate to us to a degree what happens with medication, what happens with IV lines, what happens with ventilation, but they are also very good at listening to parents I think too. (P3)

#### Morals, values and beliefs

Within the theme of *morals, values and beliefs*, *self-reflection* emerged as a key subtheme for delivering quality palliative care. Nurses reported the need to critical *self-reflection* about one’s own morals, values and beliefs when providing quality palliative care. Many expressed the need to develop an awareness of their own worldviews through ***self-reflection*** as these had the potential to impact upon their provision of palliative care. This was encapsulated by a nurse who stated “I don’t think you can force your values or put your values onto someone else but I guess your personal approach” (P5).

### Family support

Within this central theme, family support delivered by staff, the themes of *emotional support, communication,* and *practices* were identified. These factors reflected staff abilities to use their professional skills and knowledge to support families during the palliative care period.

#### Emotional support

Within this theme, 4 subthemes were identified – *attunement to family, identifying bonding opportunities, the gift of time, extended family support*. Nurses were adamant about the crucial role of emotional support for families during the palliative care period. They perceived their role to be that of facilitators of family connectedness during this distressing time. Nurses reported ***attunement*** to the family’s needs and creating opportunities for families to bond with their infants. A nurse stated simply that “these parents just need to be able to do things that they would do with their baby if it was at home” (P 4).

Nurses’ attunement to the infant and families during palliative care facilitated ***bonding*** opportunities. Understanding the needs of the infant and families allowed nurses to provide a safe environment for bonding and memory-making.

Unfortunately we knew what the outcome would eventually be but it was a matter of facilitating for that family and making sure they were supported and felt safe enough to do that on their own, and to me that’s good end of life care. (P6)

Many nurses identified the gift of ***time*** as an important aspect of providing emotional support to families. Nurses reported that families were caught between both bonding with and grieving for their baby. Nurses’ capacity to protect this time for families was perceived as important for providing quality palliative care.

I guess we’re good about privacy, we’re good about creating good moments for each of the families in their own right, and we’re good about trying to value time, because, if anything as a practitioner, if that’s what you can give them, that’s the maximum time with each other. (P2)

During this time, nurses were acutely aware of ***supporting the extended family***, as well as the parents of the infant. Often siblings, aunts, uncles, and grandparents were on the unit at the end of the infant’s life. Nurses reported that this support was crucial for immediate emotional support of the family, but also the long-term impact of this support on the family into the future.

So end of life care is about everyone, getting the whole family involved not just the parents, it’s not excluding anyone who is directly involved with that baby. (P8)

#### Communication to parents

Within this theme, four subthemes were identified – *clear information, support during decision-making, advocacy for infant, post death information*.

Nurses reported good family support was enhanced by providing ***clear information*** to families throughout the palliative care period. Clear information included being honest and truthful with families about the likely process of end-of-life for their infant, and providing education to parents to ***support the decision-making*** process. For example, one nurse reported that “I’m very honest with families. That’s a personal professional choice I make… making sure to be careful with your word choice” (P2). Another nurses stated that supporting families was enhanced “by telling them the truth [about their infant’s condition]” (P4), “empowering the parents to make…that decision at the end of the day but without forcing a particular option on them” (P5) and “the number one thing for us is to support them and help them in those decisions” (P6).

Nurses also reported that good family support was facilitated by being an ***advocate for infant***. While many nurses acknowledged the importance of the family during this period, primary nursing of the infant as the patient was also in the forefront of their minds. For example, one nurse reported, “thinking primarily is the patient in pain?…what are we asking the patient to do as far as quality of life for the time that the family needs to be able to adapt to the circumstances (P2).

Family support was also provided by nurses through provision of ***post death information***. One nurse reported that written information was important when supporting parents after the death of their infant “there’s questions and things that parents will ask, I can anticipate and I’ve already got the answers for them” (P6).

#### Practices

Within this theme, four memory-making practices were identified that were perceived to contribute to good family support during the palliative care period – ***meaning-making*** *(photographs, memento box, memory book,* and *ceremony).* Nurses also acknowledged the importance of *community support* to the provision of resources for these practices.

Nurses reported that the photographs provided by the unit were particularly important for **meaning-making** during and after the palliative care period. As one nurse described, “photographs, lots of photographs and yes just try and make the families have as best an experience they can in a bad situation” (P1). Nurses also gathered together items into memento boxes, such as locks of hair and footprints, for the families, stating “I think it’s important for that memory, the memories” (P1) “and…offering them quite a few after life memories, like the hair-clippings and memory box (P5). Nurses also spoke about their creativity in developing memory books for families about their infant. As a nurse describe “well, we’re very creative with our resources. It is nice to have the all-in-one booklet now, that we can give families as a memory” (P2). Additionally, the capacity to offer ceremony to families was also considered to be quality palliative care practice. Nurses reported that ceremonies offered to families did not necessarily have a religious affiliation. As one nurse reported, “Christenings or like name services, maybe less religious than others” (P6).

Nurses also acknowledged the **support of the community** for the unit. Community members provided hand-made items such as clothes to be given to families for their infants. Nurses reported appreciating this support as they perceived it to be a means of community acknowledgement of the difficulties of palliative care in the neonatal unit. As a nurse stated “I think and also like people in the community must realise or they’ve had it happened to them for them to make all these little dresses and then it’s so nice for the nurses to have” (P7).

### Barriers to care

**Fig. 2:**
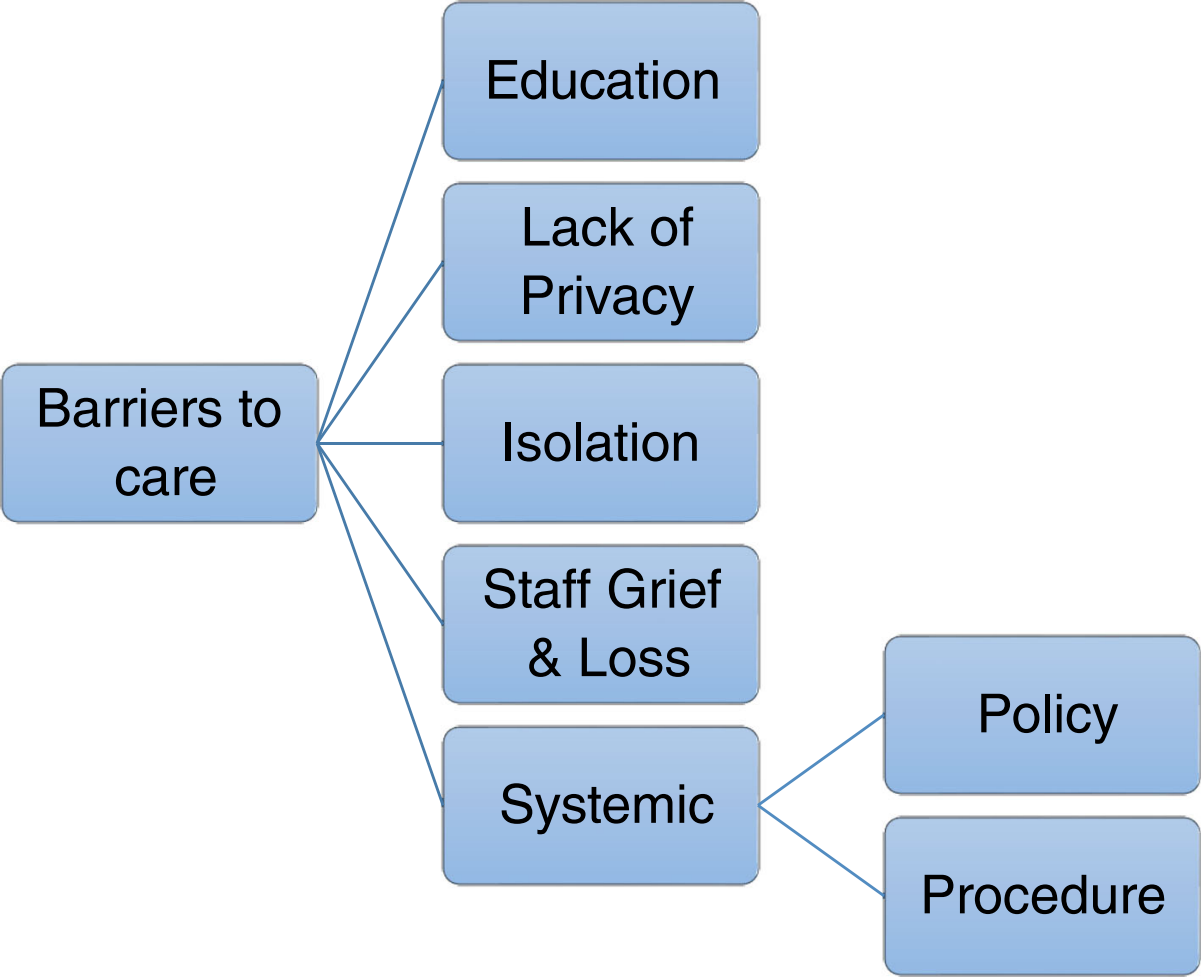
Factors nurses reported to be barriers to good care

Five themes emerged from the data that were perceived to be barriers to providing quality palliative care. Staff perceived *education, lack of privacy, isolation, staff characteristics* and *systemic (policy, and procedure)* factors impact upon palliative care provision (see Fig. 2).

#### Education

Staff perceived a ***lack of opportunities*** to engage in the palliative care process as a barrier to providing good care. Given the small number of palliative care cases in the unit, it was perceived that often the most experienced nurses were allocated to these infants and families, and that education was not provided to new staff in order to build their skills in palliative care. As one nurse noted “I think sometimes we don’t maybe educate some of the new staff well enough or involve them enough to be able to…we sort of go off the people who have maybe been there a few years and are more senior staff” (P 5) while another stated that “I’d like to know a little bit more about how we do things here…what the actual process is” (P 6). Another nurse stated that suitability and experience for providing palliative care would be enhanced “if people are more educated [so] they wouldn’t be so apprehensive about caring for babies at the end” (P6).

Further education factors included ***difficulties providing in-service*** to nursing staff. For one nurse this was viewed as a practical difficulty of releasing staff from the floor to attend workshops. This nurse stated “I’m so enthusiastic…I’ve tried to give in-services at work but it’s only people [off the floor who] can come…so availability of staff” (P8).

#### Lack of privacy

Staff perceived a barrier to care was a lack of privacy in the ***Special Care*** Unit particularly in the event of a death of a twin. For example, as nurse reported this was a difficulty when parents wanted to reunite a twin who had died in NICU with the other twin in Special Care. The nurse reported that this was particularly a difficulty for other parents in Special Care, stating,” [nurse] was taking [infant] it into Special Care to the other baby, and the parents of the other babies were getting upset because there was a dead baby in the Unit” (P4).

A lack of privacy during the palliative care period and after the death of the baby was reported by nurses particularly when parents wanted to take the dead or dying infant out of the neonatal unit into ***public spaces***. Nurses reported that parents often wanted to take their infant to the hospital gardens to experience a moment of normality. However, nurses reported that having these infants in public spaces often caused distress in others in those public spaces stating “I know that [staff have] taken babies down in to the garden [but] people get upset seeing dead babies. Or dying babies” (P4).

#### Isolation

Nurses also perceived isolation to be a barrier to care. Nurses reported that the palliative care process was often ***hidden from view*** from parents and other staff in order to ***protect*** them from the emotional distress of death and dying. As one nurse reported,

The staff, unless they’re actually involved in the end-of-life care, um, often don’t know what’s going on…because I think you try and protect other people in the unit as well. Other parents…from [the process of] dying. You’re try and protect them as well as protect parents by giving them some sort of privacy I think. (P 4)

The perception of isolation as a barrier to care was somewhat contrary to the perceptions of lack of privacy that were also reported. Negotiation of the balance between the privacy and isolation was required by nurses in order to provide quality palliative care.

#### Staff

Barriers to care were also reported to include the ***impact of nurses’ own grief and loss*** upon the delivery of care by nurses. Nurses reported that delivering palliative care was emotionally draining and required self-reflection. As one nurse reported “I’ve come to understand that that is your own personal stuff that they actually can’t deal with…grief, death and dying” (P8).

#### Systemic factors

Barriers to delivering quality palliative care included policy and procedure factors. At the ***policy*** level, nurses perceived a *lack of input into unit guidelines* for palliative care, *a lack of unit evaluation*, and the *need to update ideas and values* about care provision. As a nurse reported:

…there were six different policies…so I tried to tie them altogether, write them into one but in a more modern way and then they get shoved into the bowels of the hospital and you never see them again. (P8)

Frustrations were also expressed about the lack of input into palliative care guidelines was also reflected in the perception of a lack of evaluation for the palliative care provided. A nurse noted that “I don’t think we’ve ever evaluated ourselves” (P4). Evaluation was also perceived by nurses as a transformative process for changing guidelines and values of the unit that support palliative care. It was acknowledged that community and parents values about palliative care had changed over time and these changes were not reflected in the unit, “We’re just doing [the same palliative care], we’ve got policy and procedures, but the individuals have changed” (P4).

***Procedure*** subthemes included *lack of flexibility*, *differing levels of support*, *difficulties in skill mix* that interrupts continuity of care, and *difficulties in staff changing from model of care* from restorative to palliative care. While the policy level guidelines were perceived to be necessary for delivering quality palliative care, a barrier was perceived in the application of these into practice. A ***lack of flexibility*** in being able to apply the guidelines were perceived by nurses who reported “I think we’ve got the resources to do a lot and…we follow the physical withdrawal of care sheet and …we should not have it as a tick box but as a guide” (P5).

***Differing levels of support*** from leadership was also perceived as a barrier to providing quality palliative care. As a nurse stated, “I’ve had some great CN’s on when I’ve been facilitating and other ones that, you know want the room cleaned and ready for another baby” (P8). Further to this perceived difference in support across leadership was the perception of difficulties in care continuity due to ***skill mix***. This was particularly noted when nurses were allocated to support families after their infant had died. One nurse described her discomfort during this process given her minimal connection with the family during the palliative care period “I might have felt a bit more comfortable if I felt like I should been there more than if I’d been somebody who had more connection with that family, yeah” (P3).

The change from restorative to palliative model of care was also perceived to be a barrier from some nurses. This ***shift in model of care*** was sometimes difficult to navigate as it conflicted with the medical ideals of providing life-saving care. A nurse reported that “whereas when they’re in a palliative model…in a way totally opposite to what we normally do which is very hard for a lot of them to get their head around” (P8).

### Regional location of the unit

**Fig. 3:**
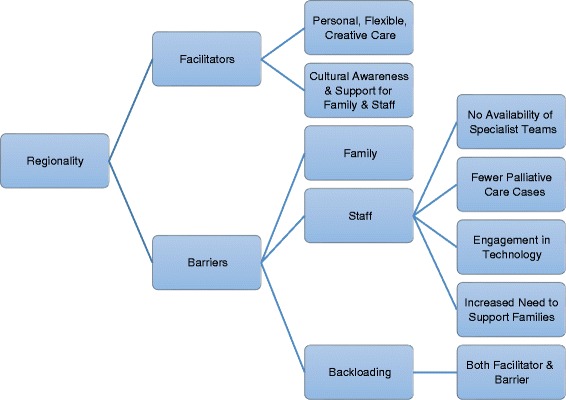
Regional location of Unit – facilitators and barriers for providing palliative care

The regional location of the unit was perceived to present both facilitators and barriers for staff and families. Half of the nurses reported that the regional location of the unit did not affect the quality of care provided by staff. One nurse summed up this perception when stating “*we’ve got the resources, we’ve got the ability to access things. I think our staff is fantastic, I don’t think there’s ever a staffing issue, I don’t think there’s a resources issue*” (P5). However, this was not the perception of all the nurses. The following outlines facilitators and barriers to providing quality palliative care for families and staff (See Fig. 3).

### Facilitators of care

Nurses perceived that being a regional unit allowed them to personalise care for families and be creative in their care. As one nurse stated, “[being a regional unit] provides us challenges for providing care. I don’t think it changes it in a negative way. I think it requires us to be more creative” (P2). These opportunities to be creative and “personalise [palliative care] for those parents” facilitated a more positive experience for families” (P8). Being a smaller regional unit “provides us a lot of variety again, because we don’t see the same clientele all the time, it does help us to be more flexible about what the family might need” (P2).

#### Cultural awareness and cultural support for staff and families

Working in a regional unit necessitated nurses to develop cultural awareness and culturally safe practices when providing care to Aboriginal and Torres Strait Islander families. Nurses described their understanding of cultural differences, stating “I think most of the Aboriginal people would prefer to have their babies with them and their families and they can’t always do that a lot…it’s just that for the parents, I think that they would probably feel more comfortable in their own environment” (P1). There was a reported awareness of the disruption this caused stating, “but if their babies are unwell they’re out of their own environment and culture, yeah cultural wishes” (P1). The regional location and concomitant cultural diversity of the unit also necessitated cultural knowledge and support from Indigenous support staff. Nurses reported that this was an important benefit to staff and families, stating, “having families that come from that far [rural and remote areas] does create challenges for us and we are definitely lucky to have things such as the Aboriginal Liaison Officer” (P2). Culturally safe practices were also important consideration, with a nurse noting that “all the culturally different people that we’ve got here so we’ve got to be culturally aware that some people do stuff different” (P7). In particular it was noted “the cultural aspects that sometimes it isn’t the parents that look after the baby, sometimes it’s a grandma” (P2).

### Barriers to care

#### Families – time pressures due to family location

Time pressure due to rural and remote locations of families was the most mentioned barrier to supporting families during palliative care. This was elegantly articulated when a nurse noted “I think sometimes it’s difficult because we are a regional, we’re a tertiary centre, a lot of our mums and dads come from far away and they can’t always have their family members with them and they’re away from home” (P1). Nurses were also required to navigate the balance between the need to attend to the baby’s medical needs and the needs for parents support. “I think if it’s a long term thing, we often encourage other family members to get here quicker” (P2). Difficulties in accommodating large extended families upon arrival to the unit were also described as a challenge. This flow-on effect was identified by a nurse who stated that “being able to accommodate families from out of town I think is a big thing” (P5).

#### Staff – availability of specialist teams

Regional location of the unit also limited the availability of specialist neonatal teams such as cardiac or paediatric palliative teams. In the event of an infant’s treatment changing from critical care to palliative care, transfer from regional to metropolitan treatment centres was reported to impact upon families. For example, “if it’s a matter of them having to fly somewhere to see that specialist team and then still being given the bad news that you can’t do anything…being regional though regardless, you don’t have all the specialities, you don’t have all the options” (P6).

#### Staff – fewer palliative care experiences

Having fewer palliative cases was identified as a challenge for staff as there were reduced opportunities for staff to develop experience and competence in delivering care. For example, a nurse reported that “it’s great you don’t have as many for the end of life but it also means staff don’t have as much experience, you don’t have as much exposure to it so that you’re not as confident and competent as maybe someone that’d be in the city where there’s a lot higher numbers, purely by ratio” (P6).

#### Staff – engage with technology

While it was identified that the unit was resourced with technology devices such as iPads, there was a perception that these were not used to their full potential to alleviate the impact of regional location upon families. It was suggested that these devices could be used to connect immediate and extended family who are separated from their infants due to distance from home. As a nurse stated “we’ve got these new iPads in the unit, why can’t we set up Skype for some of the families overseas and say look grandma do you want to say goodbye when you’re in England” (P5).

#### Staff – increased need to support families

Regional location of the unit also impacted upon the levels of support for families required from staff members. Given the time it takes for family members to arrive at the unit from rural and remote areas, staff reported needing to increase their supportive role in the interim. The impact upon staff members was described by a nurse who stated that “sometimes perhaps it’s a little more taxing on us as practitioners because maybe we do need to be a little bit more of a support group for some families because a lot of our families do come from outlying regions” (P2).

#### Transfer to local hospital dilemma – a facilitator and barrier to quality of palliative care

The practice of ‘backloading’ (transferring) infants to hospitals closer to their homes was reported as a facilitator and a barrier to palliative care. This dilemma was identified when a nurse stated,

[When we know that the baby is going to die] we like to send them back to their family. But is that a good thing? Knowing what those hospital’s resources are even more stretched than our resources. So, are we, we are in a way doing family support, but [the local hospital] have an even lesser set up [for palliative care]. I think that, than we do. That one’s [question], I don’t think, we have sent them on further, we’ve sent them back, and that’s a good thing and the parents do appreciate that, but then is that hospital set up to deal with that [dying baby]? (P4)

## Discussion

The results of this study highlight the barriers and facilitators of palliative care provision in a regional tertiary neonatal unit. The focus on both the positive and negative factors is a strength of the study that will allow a dual approach to both addressing the barriers and facilitating quality palliative care. Facilitators to care included staff factors of leadership, clinical knowledge and morals, values and beliefs, and family factors of emotional support, communication and practices. However, identified barriers were education, environment factors of lack of privacy and isolation, staff grief and loss, and systemic issues including policy and procedure factors. Barriers to care are the most commonly researched [9–13]. Few studies have included both facilitators and barriers to palliative care provision [8, 12].

Facilitators of good care identified in the current study are reflected in previous research [8, 12]. Nurses in this study identified the importance of clinical knowledge including palliative care education and the ability to adapt and tailor care to families in caring for neonates and families. These nurses reinforced the need for good clinical guidelines, communication, and evaluation of the care provided during palliative care. Establishing effective clinical guidelines provided nurses with a framework within which to deliver care. Further communication included being an advocate for the infant while supporting families to make decisions [8], and providing post-death information. Further, self-reflection upon one’s morals, values and beliefs allowed nurses to safely practise without becoming overwhelmed by the difficulties of attending to dying neonates and their families. However, staff opinions and beliefs have been previously identified as a barrier to care [9]. For these nurses, perhaps developing self-reflection has the potential to encourage staff to engage in conversations about aspects of care that are in conflict with their personal values, which may in turn help to alleviate moral distress identified by other research [13].

Strong leadership was identified by nurses in the current study as a facilitator of quality palliative care. This factor has not been explicitly identified in previous research. Strong leadership was evidenced by senior staff providing mentorship to less experienced staff, and being effective communicators in the team. Leaders in the unit were also perceived to be those nurses who were able to understand staff suitability for and experience of providing care, and being able to balance the skill mix of nursing staff. Previous research has identified inadequate staffing and moral distress to be a barrier to palliative care provision [11–13]. Supporting staff in the neonatal unit to enhance their leadership skills may help to address staffing difficulties.

Barriers to palliative care provision identified in the current study are similar to those in previous research. Attitudinal [11], educational [9, 12, 13], environmental [10–12] and institutional [9, 10] factors were identified by nurses in the current study. For example, these factors included staff grief and loss, in-service provision difficulties, isolation of and lack of privacy for families, policy guidelines and procedural flexibility. It appears that these commonly identified factors negatively impact palliative care provision in neonatal units in various countries. Further, these findings reinforce the need for effective guidelines, staff and family support, education, and evaluation of the care provided during palliative care.

As the only regional tertiary neonatal unit in Australia, it was important to also explore the impact of regional location on palliative care provision. The impact of regional location has not yet been identified in previous research. Nurses identified barriers to care relating to family and staff factors. Nurses were acutely aware of the time pressures upon families to quickly travel long distances at the end-of-life and the concomitant pressures upon staff to balance the needs of the family and the neonate. Further, staff felt the pressures of providing support to extended family members who had travelled to the unit. These pressures may be ameliorated by the use of technology to connect families during this time. For example, families could be connected to the unit using telemedicine technology such as video-link and cameras in order to see their infant.

Nurses also perceived that given the regional location of the unit, exposure to fewer palliative cases impacted upon their development of palliative care skills. Additionally, providing care to neonates without the immediate support of specialist teams was also perceived as a barrier resulting from the regional location of the unit. While development of a paediatric palliative team was proposed, given the few cases of palliative care in the regional unit, the operation of such a team was yet to be clearly defined. More creative use of technology to receive advice from subspecialists in metropolitan cities might be considered within the unit to reduce the need for babies to travel, particularly when the local care givers recognise that continued care is futile.

Given the regional location of the unit, returning neonates to their local non-tertiary referring hospitals presented a dilemma for staff. Nurses were aware that sending neonates to the local hospital would ease access for families and extended families. However, nurses were also acutely aware of the limited resources those hospitals have to provide palliative care for the dying neonate. This delicate balance was at the forefront of nurses minds when considering whether or not to transfer the neonate. Communication between the tertiary unit with the local hospitals about their willingness to provide palliative care for individual babies, facilitated by the use of telemedicine to introduce the different staff teams to the family, as well as the use of documented guidelines could facilitate timely transfer for some babies and their families to a location closer to home and community support.

Facilitators of palliative care in the current study included nurses’ perceptions of being able to provide personal, flexible and creative care for families. Nurses were able to surmount resource limitations that resulted from being geographically isolated from the nearest metropolitan area. Importantly, given that over a third of neonates in the unit were Aboriginal or Torres Strait Islander neonates, nurses reported that their level of cultural awareness helped to pave the way for culturally responsive care in the unit. There is some data available on the cultural aspects of care in the Australasian setting. Cultural specific practice explored in the New Zealand Maori population shows a desire of families and communities to be involved in palliative care planning, preferences for death to occur at home and the importance of prayer and song at the time of death [15]. Aboriginal and Torres Strait Islander peoples of Australia also have culturally important beliefs around death which need to be respected in order to provide culturally safe and supportive [14]. Connection to community may lead to a desire for collective decision making with people travelling long distances before decisions surrounding palliative care or withdrawal of care can be made.

Many Aboriginal and Torres Strait Islander people who live in small, very remote communities have strong cultural connectedness including cultural traditions and beliefs. For these Aboriginal and Torres Strait Islander families, authority over the child may not reside with the parent, and discussions need to occur with the appropriate people present. Planning for events following death may need to include the practicalities of getting the infants body back to the home area of the community – often a costly affair- and escorted by a person considered appropriate within the community. Palliative care services themselves often have a low uptake by these communities. Overall, the results of this study identified the need for connection to the area in which the family lives, and the need for cultural sensitivity in the provision of palliative care.

The strength of this study was to explore both facilitators and barriers to providing quality palliative care. The regional nature of the unit also provides a strength in exploring the perceptions in a unit where the many of the extremely sick patients are far from their community supports. There is a large component of Aboriginal and Torres Strait Islander people served by the unit, so aspects of palliative care in a culturally diverse location are investigated. The sample size is small and although this is a potential limitation of the study, this is consistent with qualitative methodology, with data saturation obtained.

## Conclusions

Previous research has predominantly focused on the barriers to providing palliative care in the neonatal environment. The strength of the current study is that it explored both facilitators and barriers to providing quality palliative care, and in doing so, makes an original contribution to the literature. Participants in this study perceived several factors, such as education, to be both a potential barrier and facilitator. Identification is the first step in a strength-based approach and implementation strategies are required to address barriers and amplify facilitating factors in order to provide quality palliative care in the neonatal context. Further, specific facilitators and barriers to palliative care provision unique to regional neonatal units, not previously explored in the literature, were identified. Study results have provided important considerations for regional and geographically isolated neonatal units, and will be used to inform clinical practice improvements, staff education support, and further research relating to palliative care provision for the most vulnerable babies and their families.
